# Prevention of Malaria in Pregnancy with Intermittent Preventive Treatment and Insecticide Treated Nets in Mali: A Quantitative Health Systems Effectiveness Analysis

**DOI:** 10.1371/journal.pone.0067520

**Published:** 2013-06-28

**Authors:** Jayne Webster, Kassoum Kayentao, Jane Bruce, Sory I. Diawara, Amadou Abathina, Alhassane Ag Haiballa, Ogobara K. Doumbo, Jenny Hill

**Affiliations:** 1 Department of Disease Control, London School of Hygiene and Tropical Medicine, London, United Kingdom; 2 Malaria Research and Training Centre, University of Bamako, Bamako, Mali; 3 Child and Reproductive Health, Liverpool School of Tropical Medicine, Liverpool, United Kingdom; 4 Programme National de Lutte Contre le Paludisme, Bamako, Mali; Tulane University School of Public Health and Tropical Medicine, United States of America

## Abstract

**Introduction:**

The objectives of the study were to evaluate the health system effectiveness of ANC for the delivery of a dose of IPTp and an ITN to women attending ANC during eligible gestation, and to identify the predictors of systems effectiveness.

**Methods:**

A cross sectional study was undertaken in 10 health facilities including structured non-participant observations of the ANC process for 780 pregnant women followed by exit interviews. The proportion of pregnant women receiving a dose of IPTp-SP and an ITN was assessed. Predictors of each ineffective intermediate process were identified using multivariable logistic regression.

**Results:**

Overall, 0% and 24.5% of pregnant women of eligible gestation on the first visit to ANC received a dose of IPTp-SP by DOT at the district and community levels respectively. Ineffective intermediate processes were ‘given IPTp-SP at the ANC’ 63.9% and 74.0% (95% CI 62.0, 83.3), and ‘given IPTp-SP by DOT’ 0% and 34.3% (95% CI 10.5, 69.8), at district and community levels, respectively. Delivery of ITNs was effective where they were in stock; however stock-outs were a problem. Predictors of receiving IPTp-SP at the district level were 4 to 6 months gestation, not reporting symptoms of malaria at ANC visit and the amount of money spent during the visit. At the community level, the predictors were 4 to 6 months gestation, maternal education below primary level, routine ANC visit (not for an illness), palpation of the abdomen, and expenditure of money in ANC.

**Conclusion:**

In Segou District, the delivery of IPTp-SP was ineffective; whilst ITN delivery was effective if ITNs were in stock. Predictors of receiving IPTp-SP at the district and community levels included gestational age, the amount of expenditure during the ANC visit and no illness.

## Introduction

Malaria in pregnancy due to *Plasmodium falciparum* is associated with maternal anaemia and low birth weight [1], [2]. Low birth weight due to malaria is preventable with intermittent preventive treatment with sulphadoxine-pyrimethamine (IPTp-SP) [3], [4]. IPTp-SP and insecticide treated nets (ITNs) are the currently recommended tools for prevention of malaria in pregnancy in sub-Saharan Africa (SSA)[5]. IPTp-SP is delivered to pregnant women primarily through antenatal clinics (ANC), and ITNs are delivered through ANC together with a variety of other systems. Based on the findings of a randomised controlled trial in 2001[6], WHO now recommend focussed ANC (fANC) [7]. As part of fANC it is recommended that a pregnant woman visits ANC four times and during these visits receive a number of interventions. These interventions aim to identify risk factors and provide prevention against the major infections causing poor outcomes in pregnancy. In areas of stable malaria transmission, until October 2012 WHO recommended that pregnant women were given 2–3 courses of IPTp-SP after the onset of foetal movement with each course at least one month apart [8]. New WHO guidelines now recommend a dose of IPTp-SP at each scheduled visit beginning as early as possible in the second trimester, and with each dose at least one month apart [9].

Despite one to two decades of implementation of IPTp-SP and ITNs within national programmes in SSA, coverage of both of these essential tools for protecting pregnant women are still low [10], [11]. As attendance at ANC at least once is high in many countries of SSA [12], [13] explanations are required as to the reason for the differences in these estimates. The most recent national level data for Mali estimated that 6.0% of pregnant women received at least one dose of SP during an ANC visit, and 4.0% received 2 doses while 71% of women attended ANC at least once and 63% attended twice [14]. Disparities across socio-economic quintiles of receipt of SP during an ANC visit were extremely high, as were attendance at ANC. This same survey estimated that 28.9% of pregnant women slept under an ITN the previous night. It is important therefore to understand the reasons that women who attend ANC do not receive IPTp-SP, so that interventions can be designed to target specific processes and to increase the effectiveness of the health system to deliver this efficacious intervention.

There have been a multitude of studies on the delivery of ITNs through the public and private sectors, through continuous and campaign delivery strategies, and on their use by target groups. However, despite delivery of ITNs through ANC being the primary continuous delivery strategy recommended by Roll Back Malaria (RBM) [15], there is very little evidence of the effectiveness of this delivery strategy. Studies have evaluated the effectiveness of delivery of ITNs through ANC via voucher schemes where the voucher subsidy is delivered in ANC and the ITN in the private retail sector [16], [17] but to our knowledge there have been no evaluations of the effectiveness of the direct delivery of ITNs themselves, through ANC.

The objectives of the study were to evaluate the health system effectiveness of ANC for delivering a dose of IPTp-SP and an ITN for women attending during eligible gestation as defined by the national policy guidelines, and to identify the predictors of systems effectiveness.

## Methods

### Ethics

The study was approved by the ethics committees of the Faculty of Medicine, Pharmacy, and Odonto-stomatology, University of Bamako, the London School of Hygiene and Tropical Medicine, and the Liverpool School of Tropical Medicine. Health workers gave signed consent at the initial meeting for structured questionnaires and for structured observations. Pregnant women gave signed consent for observations and exit interviews immediately prior to beginning the ANC process.

### Study Site

The study was conducted in Segou region located 240 kilometers East of Bamako in Mali. The region is composed of 7 districts including the district of Segou where the study took place. Segou District has a total population of 448,552 projected from the 1998 census, with more than 60% of this population living in rural areas. The climate of Segou is typical of the Sahel with an average annual precipitation of 400 mm, with a wet season of about three months (July–September) corresponding to the highest malaria transmission period [18]. There is also a ‘cold’ dry season (November–February) and a hot dry season (March–May/June) Malaria in Segou Region is seasonal ranging from holo-endemic in the southern part of the district and meso-endemic to the north.

The district has a total of 29 functioning health structures comprising 1 hospital, 1 district level health facility (*Centre de santé de reference*), and 27 community health centres (CSComs). At the time of the study there were 8 non-functional CSComs. The hospital serves as the regional referral centre and the *Centre de Santé de Reference* (CSRef) for district level referrals. The CSComs are situated between 5 km and 150 km from the CSRef. ANC services are available Monday to Friday (8.00 am–5.00 pm) in Segou District, but attendance varies by day of the week, with highest attendance often linked to market days in the closest town. Since the early 1980s a health sector reform programme facilitated the development of community-financed health centres, in a move away from a highly centralised urban bias in the health system. These community-financed health centres rely on cost recovery for the financing of most wage and non-wage recurrent costs, rather than being government financed. This means that a limited number of health staff are government funded.

### Study Design and Data Collection

A cross sectional observational study was conducted in 10 health facilities of Segou District. A dual frame sampling scheme was used to purposively select the CSRef and a representative sample of CSComs [19]. The CSRef was purposively selected as this serves as the referral centre for the district, thus sharing the total population of the district cross the CSComs. A further 9 health facilities were randomly selected using probability proportional to size from the composite list of health facilities.

Structured non-participant observations of 780 ANC visits were undertaken between 2^nd^ October and 26^th^ November 2009. The sample size was calculated using a standard method for health facility surveys to estimate proportion of women with specific service delivery endpoints [19]. The endpoints estimated were the proportion of women that were given a dose of IPTp-SP on the day of the visit, the proportion who were given SP by DOT, and the proportion that were given an ITN. A total of 305 women were needed to achieve a 5.5% precision, assuming the prevalence of the specified endpoints is 40%. The sample size was increased in order to enable the assessment of predictors of a range of processes in the delivery of IPTp-SP and ITNs, this was done by hypothetically estimating the proportion of pregnant women attending ANC who would be offered IPTp-SP, the proportion who would take the IPTp-SP when offered and the proportion who would attend a second time and therefore have the possibility of receiving a second dose. Based upon the catchment population of the CSRef being the total population of Segou District and upon the operational feasibility, one third of the sample was collected from the CSRef, with the remaining two thirds from the 9 CSComs proportional to their catchment population according to district records.

After gaining the consent of the head of each health facility, a meeting was held with all staff to inform them of the study and to collect background information from each health worker using a structured questionnaire. Written consent to be observed was obtained from health workers at this point. Five fieldworkers and three supervisors conducted the observations and interviews by approaching a woman as she entered the health facility, they then introduced the study, gained the consent of the woman, observed her ANC visit, and interviewed her on exit. On completion of the process with the first woman they then approached the next woman to enter the facility and repeated the process. The facility observation schedule was based upon local market days, and numbers of observations required at each facility. Facilities were visited by the study team mainly on the busiest ANC day, coinciding with local market day. Each facility was visited by the team until the required sample size was achieved.

Fieldworkers followed pregnant women from their entry to the health facility until their completion of the visit including various scenarios amongst registration, health education, history and vital signs, consultation, prevention of mother to child transmission of HIV (PMTCT), laboratory, post-laboratory consultation and dispensary. During these observations, fieldworkers used a structured checklist to record actions, and communications between the health provider and pregnant woman. An exit interview was conducted with the woman when ready to leave the facility with direct questions on the events during the ANC visit, observation of drugs received, and knowledge of the woman on leaving the facility. Interviews were conducted in Bambara which is the most predominant local language in Segou District.

Several different types of information were collected during the observations which included responses of pregnant women to a question by a fieldworker, responses of pregnant women to questions from a health worker during the ANC visit, actions of the pregnant women or the health worker observed by the fieldworker, communications heard by a fieldworker during observations and written information or data observed by a fieldworker.

Health facility audits were undertaken at each of the ten health facilities to assess the context within which ANC is delivered including departmental structure of the facility, size in terms of ANC attendance, numbers cadres of staff, and source of funding.

### Study Definitions

**Figure 1:**
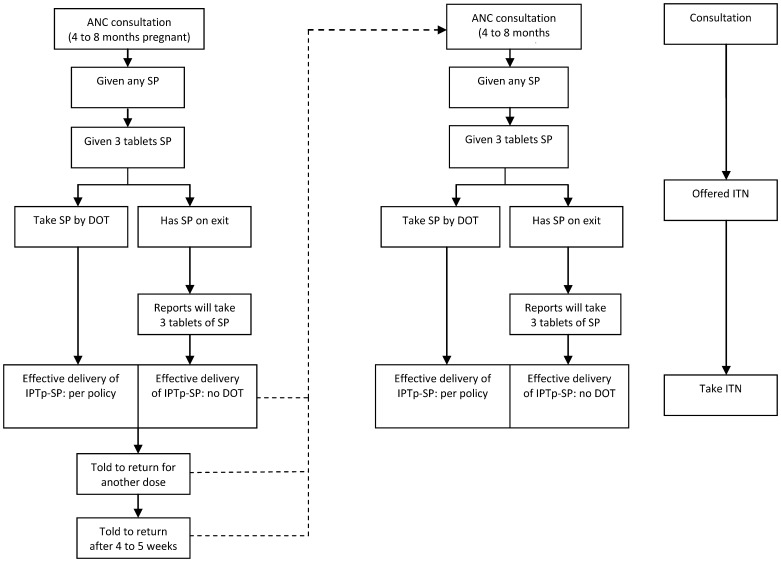
Systems effectiveness algorithm for IPTp-SP and ITNs.

A health system effectiveness algorithm was developed based upon the WHO and national policy documents on IPTp-SP and delivery of ITNs through ANC (Figure 1) which were in place at the time of data collection. Women should be given IPTp-SP twice during pregnancy in the second and third trimesters with the first dose after quickening and each dose at least one month apart [20]. According to the Malian national policy at the time of the study, IPTp-SP should be given free of charge, but should not be given to women in their ninth month of pregnancy [21]. Each of the two doses of IPTp-SP consists of 3 tablets of SP, each containing 500 mg sulpha drug and 25 mg pyrimethamine and should be taken in the facility by directly observed treatment (DOT) by the health provider.

The intermediate processes required for effective delivery of IPTp-SP per policy were therefore defined as: Intermediate process 1: attend ANC consultation between 4 and 8 months pregnant; Intermediate process 2: be given any SP; Intermediate process 3: be given 3 tablets of SP; Intermediate process 4: be given SP by DOT; Intermediate process 5: told when to return for the next visit; and Intermediate process 6 told to return in 4 to 5 weeks. The data source for each of these processes was observation of the ANC visit. In order to include the situation where a woman was not given IPTp-SP by DOT but may still have been given and taken SP, in a second analysis process 4 was defined as took the SP by DOT or left the facility with 3 tablets of SP and was able to report correctly how they would be taken. The data for these processes was obtained at exit interview. As the policy is that 2 doses of IPTp-SP should be given, full effectiveness requires that those pregnant women for whom IPTp-SP is successfully delivered once, should attend ANC a second time and progress effectively a second time through the first 4 defined processes. It is important to note that pregnant women with symptoms of malaria were not excluded from the effectiveness denominator as there is no direction on this in the national guidelines and the diagnosis of malaria in pregnancy within these health facilities needed more study. The case management of malaria in pregnancy in these health facilities will be presented elsewhere.

According to the national policy, every pregnant woman should be given an ITN on her first visit to ANC [21]. The health systems effectiveness algorithm for ITNs as included in Figure 1 is relatively simple consisting of just 3 processes: Process 1 attend ANC; Process 2 be offered an ITN; and Process 3 take the ITN.

Intermediate processes for systems effectiveness of the delivery of IPTp-SP and ITNs were described as ineffective if less that 80% of pregnant women were reached by the process [17]. Cumulative systems effectiveness represents successful coverage of women for each of the intermediate processes up to the designated point in the effectiveness algorithm.

### Analyses

Data were double entered and validated using EpiData version 3.1 [22], and Stata 11.0 [23] was used for data processing and analysis. Analyses accounted for the survey design, adjusting for clustering within health facilities. Sample weights for observations were estimated based upon the sampling probability of each selected health facility [19]. Analyses were stratified by CSRef and collated CSCom as we hypothesised that the systems effectiveness and the predictors of the intermediate processes involved in delivery of IPTp-SP would differ at these different levels of the health system.

Two systems effectiveness analyses were undertaken for IPTp-SP: 1) a cumulative analysis of the overall effectiveness of the intermediate processes for women who reported during exit interview that they were 4 to 8 months pregnant for their 1^st^and for their 2^nd^ visit to ANC, and 2) an assessment of the effectiveness of each individual intermediate process for women 4 to 8 months pregnant for their 1^st^, and 2^nd^ visits to ANC. The effectiveness of each intermediate process in the delivery system effectiveness algorithm was calculated by estimating the proportion of women who successfully reached each step from the previous step [17]. Two cumulative delivery system effectiveness estimates were calculated 1) a ‘per policy’ estimate of receiving 3 tablets of SP by directly observed therapy (DOT) on first, and second visits to ANC; 2) a with or without DOT inclusive estimate of the proportion of women either receiving 3 tablets of SP by DOT, or leaving the health facility with 3 tablets of SP and able to report on questioning that they would take all 3 tablets at one time. For ITNs the cumulative effectiveness included only 3 steps which were attend ANC (with no restrictions on gestation), offered an ITN, and given an ITN during ANC consultation.

Principal components analysis (PCA) was used to create an asset index [24], [25] based upon household characteristics such as source of drinking water, type of toilet facilities, and a range of household assets. All assets were included in the PCA as binary variables [26]. The asset index was then used to construct socio-economic quintiles from the poorest households through to the least poor. This method has been validated in household surveys with information on both assets and income or expenditure [27]. In this study these socio-economic quintiles are not representative of the population level, but are a relative score amongst women who attend ANC in Segou District, and a greater proportion of pregnant women from less poor households attend ANC in Mali [28].

Potential predictors of intermediate processes for pregnant women 4 to 8 months pregnant (for IPTp-SP) on their first visit to ANC were assessed using a univariate (unadjusted) logistic regression model. Categories of potential predictors included: socio-demographic; pregnancy factors; health facility factors; and 3 types of process factors which were departments and time; illness or suspected illness; and payments. Individual potential predictors within each of these categories were measured during the structured observations by fieldworkers observing actions, listening to communications, or observing written information (Table 1).

**Table 1:** Descriptions of the potential predictors included in the univariate analyses.

Indicator	Description	Source
**Pregnant women socio-demographic**		
Age group	Age	1
Marital status	Single (incl. engaged), married, divorced	1
Education	‘Formal’ with primary lowest level	1
Ethnicity	6 named response categories+other	1
Religion	3 named response categories+other	1
Socio economic status	Questions on a range of household assets	1
**Pregnancy factors**		
Months pregnant	On the day of the survey	1
Number of children	Live children only	1
Visit number	Number of visits to ANC including the current one	1
**Health facility factors**		
Cadre of health worker in consultation	Based on previous enrolment of health workers	3
**Process factors:**		
**1. departments and time**		
Morning consultation	Consultation between 8 am and 12 pm	3
Palpation	Palpation of the abdomen in ANC consultation	3
Prescribed rhesus test	Prescribed during ANC consultation	4+/−5
Total time spent in consultation	From entry to completion of ANC consultation	3
Had HIV PMTCT consultation	Entered the designated room and was seen by a health worker and/or HIV was discussed during ANC	3
Given ITN in consultation	Was offered and took an ITN	3
**2. illness or suspected illness**		
Reason for visit	Routine ANC visit alone, or also because of illness	1
Suggest a lab test during consultation	Any lab test suggested during ANC consultation	4+/−5
Prescribed syphilis test	During ANC, or PMTCT consultations	4+/−5
Report illness at consultation	Any illness reported by the woman during consultation	2
Consult malaria	Woman reports that she has malaria at consultation	2
Has symptoms of malaria	Woman reports that she has malaria, fever, shivers, or chills at ANC	2
Woman reports symptoms of STI/UTI	Woman reports vaginal discharge, dysuria or UTI at ANC	2
HIV in PMTCT	Tested for HIV in PMTCT	2
**3. payments**		
Pay for registration	Any money paid in registration	3+/−5
Pay for consultation	Any money paid in consultation	3+/−5
Pay for medicines	Any money paid for medicines	1
Amount paid for medicines	Total amount paid for medicines	1
Spent money on travel	Any money spent on travel to and from the facility	1
Amount spent on travel	Amount of money spent on travel to and from the facility	1
Money expenditure in the facility	Any money spent in the facility during this visit	1
Total expenditure	Total amount of money spent in the facility during this visit	1

Source Key.

1 = response of pregnant woman to a question by a fieldworker; 2 = response of pregnant woman to a question from a health worker; 3 = action observed by fieldworker; 4 = heard by a fieldworker during observations; 5 = written information/data observed by fieldworker; PMTC, Prevention of Mother to Child Transmission; ITN, Insecticide Treated Net; STI, sexual transmitted infection; UTI, Urinary Tract Infection; HIV, human immunodeficiency virus; ANC, Antenatal clinic;

Potential predictors were analysed for those intermediate processes that were found to be ineffective, that is for which less than 80% of women who should have completed the process, did so. These analyses were restricted to women 4 to 8 months pregnant, but gestation was included as a potential predictor. Adjusted Wald tests were used to assess the association between each potential predictor and the outcome of each intermediate process. Predictors with Odds Ratios (ORs) significant at the 10% level (p-values<0.1) were included in multi-variable (adjusted) logistic regression models for each intermediate process outcome in order to determine which potential predictors remained associated with each of the outcomes when adjusted for other predictors. ORs were estimated rather than Relative Risk (RR) due to clustering and stratification of the sample, and therefore our outcomes not being truly representative of the district level population. In the multi-variable models, predictors were considered significant at the 10% level at all stages of model building except for the final model where p<0.05 was used [14].

The design effect (DE) and intra-cluster correlation coefficients (ICC) were calculated for 6 systems effectiveness intermediate processes defined in the health systems effectiveness algorithm (Figure 1) for aggregated CSRef and CSComs, and for aggregated CSComs alone. The design effect was calculated as the difference between the variance based on the clusters used compared with a modelled variance if a simple random sample was used. A design effect of 1 means that variance is unaffected by clustering and is equivalent to that of a simple random sample. An ICC of 0 equates to a DE of 1 where the variance is equivalent to that of a simple random sample.

## Results

### Characteristics of the Health Facilities

The sample included 1 CSRef, 8 CSComs and 1 Dispensary for ease of presentation we included the dispensary amongst the CSComs in this paper (Table 2). Eight facilities had at least one staff member funded by the government. Cadres of staff with government salaries included: doctors, midwives, nurses, public health nurses, health technicians, and auxillaries. The number of ANC attendees during 2008 ranged from 165 in the Dispensary to 2,811 in the CSRef. The number of staff usually in ANC ranged from 2 to 7. The CSRef and two of the CSComs had a functioning laboratory, with malaria microscopy performed in the CSRef and one of the CSComs. All facilities had a pharmacy with the exception of one CSCom and the Dispensary. Numbers of structured observations by facility are presented in Table 2.

**Table 2:** Characteristics of health facilities.

	1	2	3	4	5	6	7	8	9	10
Level	CSRef	CSCom	CSCom	CSCom	CSCom	Dispensary	CSCom	CSCom	CSCom	CSCom
No. Staff in ANC	6	4	3	3	2	3	2	3	3	7
Have lab	yes	no	no	No	No	no	yes	no	no	yes
Have a pharmacy	yes	yes	yes	yes	yes	no	no	yes	yes	yes
ANC registrants^1^*	1,039	880	841	756	700	105	318	757	381	1,279
ANC attendees^2^*	2,811	1,871	1,938	1,642	1,349	165	637	1,302	714	1,885
Distance from CSRef (Km)	–	5	50	45	120	15	10	150	20	5
No. Of observations	259	96	43	60	88	64	48	44	47	29

1 attending for the first time during the current pregnancy;

2 attending for the first time or multiple times during the current pregnancy; *during 2008.

CSRef; Reference Health Centre; CSCom, Community Health Centre; ANC, antenatal clinic; Km, kilometre;

No., number.

There were no stock-outs of SP in any of the sampled health facilities during the period of data collection. The CSRef and two of the CSComs had a stock-out of ITNs during the data collection period.

### Characteristics of Health Workers

A median of 4 (range 1–10) health workers was interviewed per facility, with a total of 68 across all 10 facilities. The health workers were 32.4% male and 67.6% female, had a mean age of 34 (range 20–57) and were from 12 ethnic groups: 41.0% Bambara, 14.7% Malinke, 11.8% Peuhl (Fulani), 10.0% Minianka, and less that 10.0% Senoufo, Bobo, Arabe, Bozo, Dogon, Maure, Sarakole and Sonrhai. A total of 61.8% of health workers were from Segou Province, 52.9% from Segou District, and 32.4% were from the town or village within which they were providing health care. Nearly a quarter (23.5%) of those interviewed had worked in the current health facility for less that 1 year, 23.5% for 1 to 3 years, and 53.0% for more than 3 years. All health workers interviewed support the care of pregnant women and 57.4% worked in ANC. Amongst the ANC consultations observed, 47.7% were conducted by a ‘*matrone*’ (an under qualified nurse), 35.4% by trainee/non-permanent cadres (midwives or obstetric nurses), 10.4% by qualified obstetric nurses (trained in nursing and obstetrics), and 6.5% by qualified midwives (not trained in nursing).

### Characteristics of Pregnant Women

A total of 780 ANC visits were observed, and 770 pregnant women interviewed on exiting the health facility. The population of pregnant women attending ANC in the CSRef varied in several indicators compared with those attending the CSComs (Table 3). Women attending the CSRef were generally younger, had a higher level of education, were more ethnically diverse, of higher socio-economic status, had lesser number of children or were primigravidae and were more likely to be attending ANC for a routine visit together with an illness.

**Table 3:** Characteristics of pregnant women attending the CSRef and the CSComs.

Characteristic	CSRef (N = 259)		CSComs (N = 521)		
	n	%	n	%	p
Age group					0.04
<20	67	25.9	121	21.9	
20–29	135	52.1	247	47.8	
30–39	51	19.7	139	27.4	
40–49	6	2.3	14	2.9	
Education					0.004
None	90	34.7	292	56.3	
Primary	106	40.9	198	39.2	
Primary +	63	24.3	31	4.5	
Marital status					0.31
Married	229	89.1	469	92.5	
Single	27	10.5	44	7.3	
Divorced	1	0.4	1	0.2	
Ethnic group					0.01
Bambara	100	38.6	271	52.9	
Peuhl (Fulani)	40	15.4	73	15.4	
Other	119	45.9	177	31.7	
Religion					0.44
Muslim	253	98.4	499	96.9	
Christian	4	1.6	10	1.8	
Other	0	–	7	1.3	
SES group					0.0002
1 poorest	6	2.3	147	28.2	
2 very poor	19	7.4	134	27.4	
3 poor	37	14.5	118	24.8	
4 less poor	90	35.2	61	12.0	
5 least poor	104	40.6	48	7.6	
Number of Children					0.0001
0	75	29.0	119	21.6	
1	73	28.2	98	18.9	
2–4	85	32.8	236	45.5	
5+	26	10.0	68	14.1	
Gestational age					0.16
1–3 months	39	16.9	56	11.6	
4–6 months	100	43.3	205	43.3	
7–9 months	92	39.8	199	45.1	
ANC Visit number					0.06
1	110	42.5	280	51.2	
2	66	25.5	133	26.2	
34≥5	413111	15.812.04.2	622521	13.45.04.3	
Reason for visit					0.006
Routine ANC	188	72.6	417	80.5	
Routine ANC+ill	71	27.4	98	19.5	

Notes:

Primary+ = any level above primary (secondary, tertiary etc).

CSRef, Reference Health Centre; CSCom, Community Health Centre; n, number of events; N, sample size; ANC, Antenatal clinic;

### Systems Effectiveness of Delivery of IPTp-SP

**Figure 2:**
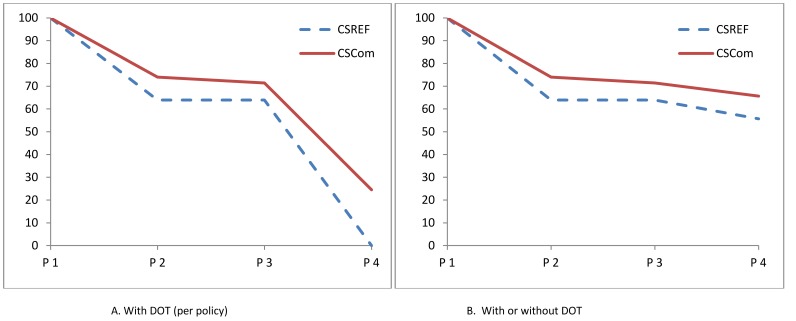
Cumulative effectiveness of delivery of IPTp during 1^st^ ANC visit by women 4 to 8 months pregnant. Intermediate process 1 = attend ANC consultation; Intermediate process 2 = given SP during ANC consultation; Intermediate process 3 = given 3 tablets SP; Intermediate process 4 = take SP by DOT (A); or take SP by DOT or have 3 tablets on exit and can report correctly how they will be taken (B).

The cumulative systems effectiveness for receiving one dose of IPTp-SP by DOT (processes 1 to 4), for women attending CSRef was 0% on their first visit to ANC and 2.1% on their second visit (Figure 2). Amongst pregnant women attending CSComs for their first ANC and second ANC visits 24.5% and 25.4% received IPTp-SP by DOT, respectively (Table 4). For women on their third visit to ANC the systems effectiveness of receiving one dose of IPTp-SP by DOT for women attending the CSRef was 0% and 8.5% for women attending the CSComs.

**Table 4:** Cumulative and intermediate process effectiveness of delivery of IPTp-SP amongst those 4 to 8 months pregnant.

	1^st^ visit							2^nd^ visit						
	CSRef			CSCom				CSRef			CSCom			
		Intermed	Cum		Intermed		Cum		Intermed	Cum		Intermed		Cum
	n	%		n	% (95% CI)	p		n	%		n	% 95% CI	p	
**With DOT**														
Attended ANC	61			189				48			109			
Given SP during consultation	39	63.9	63.9	141	74.0(62.0, 83.3)	0.09	74.0	25	52.1	52.1	81	72.4 (49.6,87.5)	0.07	72.4
Given 3 tablets	39	100.0	63.9	136	96.5(89.8, 98.8)	0.59	71.4	25	100	52.1	79	97.1 (88.5,99.3)	0.80	70.4
Took SP by DOT	0	0	0	55	34. 3(10.5, 69.8)	0.36	24.5	1	4	2.1	31	36.0 (10.4,73.3)	0.001	25.4
**Without DOT**														
Attended ANC	61			189				48			109			
Given SP during consultation	39	63.9	63.9	141	74.0(62.0, 83.3)	0.09	74.0	25	52.1	52.1	81	72.4 (49.6,87.5)	0.07	72.4
Given 3 tablets	39	100.0	63.9	136	96.5(89.8, 98.8)	0.59	71.4	25	100	52.1	79	97.1 (88.5,99.3)	0.59	70.4
Has SP tablets on exit	35	89.7	57.4	74	59.5(27.4, 85.1)	0.01	44.3	24	96	50.0	45	60.7 (26.9, 86.6)	0.004	42.7
Report will take 3 tablets	34	97.1	55.7	72	97.0(90.3, 99.1)	0.90	42.0	21	87.5	43.8	45	100	0.004	42.7
**With or without DOT inclusive**														
Report will take 3 tablets & has tabletson exit or took SP by DOT	34	87.2	55.7	127	91.9(82.2, 96.5)	0.23	66.7	22	88	45.8	76	96.7 (83.9, 99.4)	0.08	68.1
Told when to return for next dose	8	23.5	13.1	27	18.8(9.3, 34.5)	0.44	12.3	1						
Told to return in 4 to 5 weeks	8	100	13.1	24	88.2(61.7, 97.2)	0.51	10.8	1						

Notes:

CSRef, Reference Health Centre; CSCom, Community Health Centre; n, number of events; CI, Confidence Interval; Cum, cumulative process; Intermed, Intermediate process; DOT, Direct Observed Treatment; ANC, Antenatal Clinic; SP, Sulphadoxine-pyrimethamine.

Where the definition of systems effectiveness was broadened to include both those women who received IPTp-SP by DOT, and those who have 3 tablets on exiting the health facility and are able to report correctly how to take them, then the systems effectiveness was increased. For pregnant women attending the CSRef cumulative systems effectiveness for processes 1 to 4 increased to 55.7% for those on their 1^st^ visit, 45.8% for those on their 2^nd^ visit, and 37.9% for those on their third visit; for the CSComs 66.7% on the 1^st^ visit, 68.1% on the 2^nd^ visit and 45.7% on the third visit. These are likely to be overestimates as they assume that 100% of pregnant women adhere to taking all 3 doses outside of the health facility.

Estimating the intermediate process systems effectiveness in delivery of one dose of IPTp-SP based upon the algorithm presented in Figure 1, two intermediate processes in the delivery of IPTp-SP per policy were ineffective which were 1) being given SP in ANC, and 2) being given SP in ANC by DOT. Approximately two thirds of pregnant women (63.9%) who were between 4 and 8 months pregnant when they attended the CSRef for their first ANC visit were given SP, whilst three quarters of eligible pregnant women (74.0%; 95% CI 62.0, 83.3) were given SP on their 1^st^ visit to the CSCom (Table 3). The majority of women at the CSRef and CSComs who were given SP, correctly were given 3 tablets on their first visit. Amongst pregnant women attending ANC and receiving SP at the CSRef none were given the SP under DOT, whilst amongst those attending a CSCom 34.3% (95% CI: 10.5, 69.8) were given the SP by DOT.

On completing ANC consultation, pregnant women on their 1^st^ and 2^nd^ visits should be told that they need to return to ANC, and they should be told when to return. The intermediate process of being told to return was ineffective, with 23.5% and 4.5% of those attending the CSRef, and 18.8% and 8.6% of those attending the CSComs being told to return at the close of their 1^st^ and 2^nd^ visits, respectively. For those on their 1^st^ visits to both CSRef and the CSComs who were told to return, the time within which they were told to return was correct for greater than 80% in both categories of facility, and therefore an effective intermediate process according to the definition adopted for this study.

### Systems Effectiveness of Delivery of ITNs

**Figure 3:**
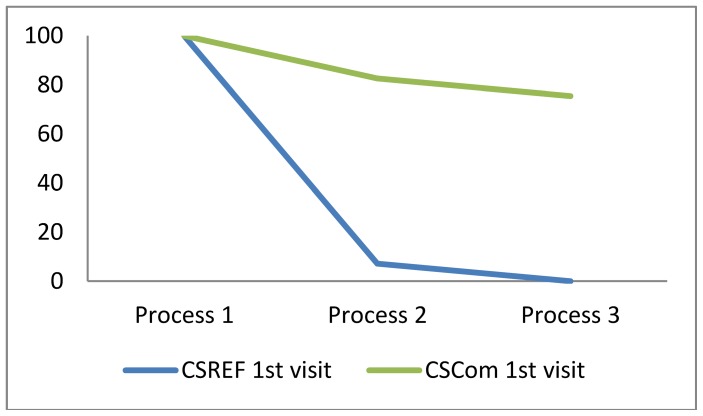
Cumulative effectiveness of delivery of ITNs during 1^st^ ANC visit by women of any gestation. Intermediate process 1 = attend ANC; Intermediate process 2 = offered ITN during ANC consultation; Intermediate process 3 = take ITN.

The CSRef did not have ITNs in stock during the period of data collection, therefore none of the pregnant women attending the CSRef for their ANC visit of any gestation were given an ITN during the survey (Figure 3) and the health systems effectiveness was 0%. Eight women reported that they were offered an ITN even though there were stock-outs. The cumulative effectiveness of delivery of ITNs through the CSComs for women of any gestation on their 1^st^ visit was 72.4%, and amongst those on their 2^nd^ visit 5.6% (Table 4).

The systems effectiveness algorithm for delivery of ITNs used in this study included only 3 intermediate processes the first of which, attendance at ANC provided the denominator for the estimates of other intermediate processes. For women attending ANC in the CSComs the offer of an ITN by health workers was just below the defined effectiveness target of 80% (Table 5). The uptake of ITNs amongst those women offered it on a 1^st^ ANC visit was effective in this group of women, 91.2%.

**Table 5:** Cumulative and intermediate process effectiveness of delivery of ITNs amongst pregnant women of any gestation.

	1^st^ visit							2^nd^ visit						
	CSRef			CSCom				CSRef			CSCom			
		Intermed	Cum		Intermed		Cum	Intermed		Cum		Intermed		Cum
	n	%		n	%(95% CI)	p		n	%		n	% 95% CI	p	
**Attended ANC**	110			280				66			133			
**Offered ITN during consultation**	8	7.3	7.3	232	79.4(55.9, 92.2)	<0.0001	79.4	0	0	0	10	6.5 (1.8, 21.1)	–	6.5
**Given ITN during consultation**	0	0	0	211	91.2(63.1,98.4)	–	72.4	0	0	0	10	86.2 (1.0, 100)	–	5.6

Notes:

CSRef, Reference Health Centre; CSCom, Community Health Centre; n, number of events; CI, Confidence Interval; ANC, Antenatal Clinic; ITN, Insecticide Treated Net.

Intermed = Intermediate process effectiveness; Cum = cumulative effectiveness.

### Predictors of being given SP

In the unadjusted logistic regression analyses for women attending the CSRef predictors of being given SP included 2 pregnancy related and 9 process related factors (Table 6).

**Table 6:** Unadjusted and adjusted predictors of receiving SP during consultation.

Potential predictors	CSRef					CSCom				
	Unadjusted			Adjusted		Unadjusted			Adjusted	
	n	OR (95% CI)	p	OR (95% CI)	p	n	OR (95% CI)	p	OR (95% CI)	p
**Socio-demographic**										
**Education level**										
None	55	1.0	0.31			199	1.0	**0.02**	1.0	<0.001
Primary	74	0.93 (0.46, 1.88)				136	1.42 (0.64, 3.15)		1.56 (0.57, 4.26)	
Primary +	37	1.70 (0.73, 3.93)				19	0.37 (0.13, 1.03)		0.25 (0.16, 0.41)	
**Pregnant woman**										
**Months pregnant**										
4–6 months	100	1.0	**<0.001**	1.0	0.002	205	1.0	**0.003**	1.0	0.03
7–8months	66	0.24 (0.12, 0.48)		0.24 (0.09, 0.60)		149	0.25 (0.12, 0.56)		0.34 (0.13, 0.88)	
**Visit number**										
3	61	1.0	**<0.001**	1.0	0.22	54	1.0	**0.04**	1.0	0.26
2	48	6.65 (2.92, 15.15)		2.14 (0.29, 16.15)		109	4.38 (1.54, 12.41)		2.05 (0.66, 6.33)	
1	57	4.08 (1.74, 9.56)		2.47 (0.89, 6.88)		189	4.04 (1.28, 12.75)		3.03 (0.81, 11.34)	
**Process factors**										
**Time in consult (minutes)**	162	1.04 (1.01, 1.08)	**0.03**	1.04 (0.99, 1.08)	0.11	348	0.99 (0.98, 1.00)	0.17		
**Went to PMTCT**										
No	150	1.0	**0.02**	1.0	0.16	313	1.0	0.92		
Yes	16	4.03 (1.24, 13.08)		2.65 (0.68,10.27)		41	0.95 (0.32, 2.85)			
**Reason for visit**										
ANC	121	1.0	**0.009**	1.0	0.13	290	1.0	**0.06**	1.0	0.04
ANC & illness	45	0.37 (0.18, 0.78)		0.49 (0.20, 1.22)		63	0.52 (0.26, 1.03)		0.39 (0.16, 0.97)	
**Symptoms malaria**										
No	145	1.0	**0.04**	1.0	0.08	306	1.0	0.29		
Yes	21	0.33 (0.11, 0.94)		0.33 (0.09, 1.13)		48	0.66 (0.29, 1.51)			
**Palpate**										
No	44	1.0	0.27			92	1.0	**0.07**	1.0	0.04
Yes	120	1.49 (0.73, 3.01)				260	1.54 (0.97, 2.46)		1.67 (1.03, 2.71)	
**Suggest a lab test**										
No	107	1.0	**0.003**	1.0	0.58	313	1.0	0.16		
Yes	58	2.74 (1.42, 5.30)		1.77 (0.24, 13.17)		39	1.51 (0.82, 2.78)			
**Prescr syphilis test**										
No	110	1.0	**<0.001**	na		328	1.0	0.26		
Yes	56	3.15 (1.61, 6.16)				26	1.57 (−.67, 3.72)			
**Prescr rhesus test**										
	114	1.0	**0.002**	1.0	0.62	325	1.0	0.33		
	52	2.87 (1.45, 5.66)		0.59 (0.07, 4.81)		29	1.23 (0.78, 1.95)			
**Pay for registration**										
No	105	1.0	**<0.001**	1.0	0.62	176	1.0	**0.07**	1.0	0.40
Yes	61	3.26 (1.69, 6.29)		0.58 (0.07, 4.90)		178	1.80 (0.95, 3.41)		0.82 (0.50, 1.36)	
**Spent any money**										
No	1	na				62	1.0	**0.05**	1.0	0.07
Yes	165					292	2.23 (1.01, 4.93)		2.16 (0.91, 5.14)	
**Total money spent**										
<500	79	1.0	**<0.001**	1.0	0.02	69	1.0	0.19		
500–999	51	4.89 (2.27, 10.49)		2.57 (0.66, 9.94)		80	2.27 (0.71, 7.33)			
≥1000	35	1.36 (0.60, 3.09)		0.56 (0.17, 1.86)		143	1.32 (0.62, 2.84)			
**Given ITN**										
No	165	na				203	1.0	**0.04**	1.0	0.17
Yes	1					151	2.68 (1.08, 6.67)		2.01 (0.69, 5.86)	

Notes:

CSRef, Reference Health Center; CSCom, Community Health Cebter; OR, Odds Ratio; CI, Confidence Interval; n, number of events; PMTCT, Prevention of Mother to Child Transmission; ANC, Antenatal Clinic; ITN, Insecticide Treated Net; Prescr, prescription; na, not applicable;

In the multivariate analysis only three predictors remained significantly associated with the systems effectiveness of being given SP. They were gestational age, had no symptoms of malaria, and the total expenditure in the health facility. A final model including these 3 indicators alone found that women who attended ANC at the CSRef were more likely to be given SP if the total expenditure they reported during the visit to ANC was between CFA 500 to 999 (adjusted OR 3.1 95% CI 1.4, 7.2; p = 0.006) compared to CFA <500 spent; and less likely to receive IPTp-SP if they were 7 to 8 months pregnant (adjusted OR 0.23 95% CI 0.11, 0.5; p = 0.002) than if they were 4 to 6 months pregnant. Women were less likely to receive IPTp-SP if they reported symptoms of malaria during ANC consultation (adjusted OR 0.27 95% CI 0.09, 0.84; p = 0.02).

The predictors of being given SP in the CSComs differed from those of the CSRef (Table 5). In the univariate analyses, there was 1 socio-demographic, 2 pregnancy related and 5 process predictors of being given SP in a CSCom.

When adjusted for other univariate predictors, those multivariate predictors remaining significant were the education level of the pregnant woman, gestational age, the reason for the visit, palpation, and any expenditure during the visit. A final model (data not shown) including these 5 indicators showed that women who attended ANC at the CSComs were more likely to be given SP if they were palpated in ANC consultation (adjusted OR 1.77 95% CI 1.0, 3.12; p = 0.05) or had any expenditure in the health facility during the visit (adjusted OR 2.27 95% CI 0.99, 5.2; p = 0.05); and less likely to be given IPTp-SP if they were educated above primary level (adjusted OR 0.28 95% CI 0.18, 0.45; p = 0.0001) were 7 to 8 months pregnant (adjusted OR 0.25 95% CI 0.11, 0.6; p = 0.005), or attended ANC for a routine visit and because they were ill (adjusted OR 0.4 95% CI 0.17,0.97; p = 0.04).

### Predictors of being given SP by DOT

Women who attended ANC at the CSRef were not given SP by DOT. Amongst pregnant women who were given SP at ANC in a CSComs, unadjusted predictors of being given SP by DOT included 1 socio-demographic factor, and 5 process factors (Table 7).

**Table 7:** Unadjusted and adjusted predictors of being given SP by DOT amongst those who were given IPTp-SP in consultation at CSComs.

Potential predictors	SP DOT				
	Unadjusted			Adjusted	
	n	OR (95% CI)	p	OR (95% CI)	p
**Socio-demographic**					
**SES group**					
1 (poorest)	109	1.0	**0.02**	1.0	0.04
2 (very poor)	85	1.09 (0.41, 2.89)		1.16 (0.30, 4.48)	
3 (poor)	84	0.37 (0.13, 1.10)		0.38 (0.10, 1.50)	
4 (less poor)	41	0.29 (0.05, 1.70)		0.47(0.12, 1.81)	
5 (least poor)	29	0.20 (0.02, 2.32)		0.45 (0.34, 5.97)	
**Process factors**					
**Suggest a lab test**					
No	313	1.0	**0.07**	1.0	0.71
Yes	39	0.20 (0.03, 1.14)		1.67 (0.77, 36.48)	
**Presr syphilis test**					
No	328	1.0	**0.03**	1.0	0.16
Yes	26	0.07 (0.06, 0.78)		0.17 (0.01, 2.44)	
**Pay for consult**					
No	274	1.0	**0.03**	1.0	0.09
Yes	80	0.07 (0.01, 0.48)		0.12 (0.01, 1.52)	
**Money is spent**					
No	62	1.0	**0.09**	na	
Yes	292	0.36 (0.11, 1.21)			
**Total money spent**					
<500	69	1.0	**0.004**	1.0	0.01
500–999	80	12.57 (2.25, 70.06)		9.87 (1.28, 75. 71)	
≥1000	143	18.17 (5.10, 64.75)		12.17 (3.14,47.16)	

Notes:

SP, Sulphadoxine-pyrimethamine; DOT, Direct Observed Treatment; n, number of events; OR, Odds Ratio, CI, Confidence Interval; SES, Socio-economic Status; Na, not available.

When adjusted for other univariate predictors, the only multivariate predictors remaining significant were socio-economic group of the pregnant woman, and the total expenditure in the health facility on the day of the visit. In the final model (data not shown), pregnant women were more likely to be given SP by DOT if they spent 500 to 999CFA (adjusted OR 9.87 95% CI 1.28, 75.71; p = 0.01) or ≥1,000 CFA (adjusted OR 12.17 95% CI 3.14, 47.16; p = 0.01) than if they spent less than 500CFA.

### Predictors of Receiving an ITN

Women who attended ANC at the CSRef for their first ANC visit were not offered and were not given an ITN as there was a stock-out at the time of the study. Amongst women attending ANC at the CSComs there was 1 socio-demographic, and 7 process factors that were predictive of being offered an ITN in ANC in univariate analyses (Table 8).

**Table 8:** Unadjusted and adjusted predictors of being offered an ITN in the CSComs.

Potential predictors	ITNs				
	Unadjusted			Adjusted	
	n	OR (95% CI)	p	OR (95% CI)	p
**Socio-demographic**					
**SES group**					
1 (poorest)	61	1.0	**0.008**	1.0	0.002
2 (very poor)	54	0.55 (0.28, 1.06)		0.37 (0.14, 0.97)	
3 (poor)	42	1.19 (0.26, 5.34)		5.74 (0.14, 229.2)	
4 (less poor)	13	0.25 (0.53, 1.13)		0.06 (0.02, 0.17)	
5 (least poor)	16	0.37 (0.02, 6.64)		3.15 (0.36, 27.21)	
**Process factors**					
**Reason for visit**					
ANC	148	1.0	0.02	1.0	0.24
ANC & illness	40	0.48 (0.26, 0.89)		0.41 (0.08, 2.05)	
**Report malaria**					
No	159	1.0	0.07	1.0	0.04
Yes	30	0.56 (0.30, 1.07)		17.71 (1.24, 252.1)	
**Palpate**					
No	60	1.0		1.0	0.02
Yes	127	3.78 (1.63, 8.77)	0.006	4.62 (1.35, 15.75)	
**HIV at PMTCT**					
No	171	1.0	0.05	1.0	0.07
Yes	18	0.07 (0.01, 0.95)		0.04 (0.001, 1.47)	
**Pay for consult**					
No	154	1.0	0.04	1.0	0.01
Yes	35	0.11 (0.01, 0.85)		0.07 (0.01, 0.43)	
**Money spent on travel**					
	129	1.0	0.05	1.0	0.32
	31	0.93 (0.18, 4.66)		0.96 (0.09, 10.14)	
	27	0.33 (0.14, 0.75)		0.14 (0.01, 1.91)	
**Total money spent**					
<500	8	1.0	0.05	1.0	0.002
500–999	49	–		–	
≥1000	117	5.8 (0.97, 34.57)		8.79 (2.74, 28.23)	

Notes:

n, number of events; OR, Odds Ratio, CI, Confidence Interval; SES, Socio-economic Status; ANC, Antenatal Clinic; HIV, Human Immunodeficiency Virus; PMTCT, Prevention of Mother to Child Transmission.

When adjusted for other univariate predictors, those multivariate predictors remaining significant were the socio-economic status of the pregnant woman’s household, symptoms of malaria, palpation, paying money during consultation, and the total expenditure at the health facility. A final model (data not shown) including these adjusted predictors of being offered an ITN in ANC showed that pregnant women were more likely to be offered an ITN if they were palpated (adjusted OR 5.0 95% CI 1.3, 19.5 p = 0.03); and if the total expenditure in the health facility was ≥CFA1,000 (adjusted OR 10.3 95% CI 2.7,39.5; p = 0.004). They were less likely to be offered an ITN if they were from the 4^th^ socio-economic quintile (adjusted OR 0.08 95%CI 0.04, 0.16), that is less poor households; and if they paid money for consultation (adjusted OR 0.05 95% CI 0.007, 0.41; p = 0.01).

### Design Effect and Intra-cluster Correlation

The within cluster correlation was low for being told to return for their next visit in 4 to 5 weeks amongst pregnant women in the CSComs who were told to return at all (DE 0.58, ICC −0.007), for this indicator the sample therefore resembled that of a simple random sample (Table 9). For indicators of relatively effective processes such as being given 3 tablets of SP amongst those given any SP (97.1%; DE 1.49, ICC 0.009), being told to take 3 tablets (93.4%; DE 1.07, ICC 0.001) and being able to report on exit that they would take 3 tablets at one time, amongst those with 3 tablets (98.5% DE 0.83, ICC −0.003) as would be expected due to the high coverage across facilities clustering within health facilities was low. Amongst the ineffective processes being given SP by DOT was found to cluster more by health facility (33.1% DE 21.11, ICC 0.353) than receiving any SP during consultation (68.0% DE 2.86, ICC 0.033) and being told when to return for the next dose (14.8% DE 2.53, ICC 0.027).

**Table 9:** Design effect and intra cluster correlation of delivery processes amongst the CSRef plus CSComs and the CSComs.

Delivery process	CSCom			CSCom & CSRef		
	% pregnant women(range)	DE	ICC	% pregnant women (range)	DE	ICC
**Attended ANC**	100	–	–	100 (−)	–	–
**Given SP during consultation**	68.0 (40.6–78.9)	2.86	0.033	60.9 (40.6–78.9)	8.08	0.139
**Given 3 tablets**	97.1 (92.3–100)	1.49	0.009	97.5 (90–100)	1.4	0.008
**Took SP by DOT**	33.1 (0–98.3)	21.11	0.353	25.3 (0–98.3)	26.1	0.492
**Told to take 3 tablets**	93.4 (0 100)	1.07	0.001	93.4 (0–100)	1.07	0.001
**Has SP tablets on exit**	61.8 (1.8–95.5)	18.22	0.302	69.1 (1.8–95.4)	21.2	0.396
**Report correctly how to take tablets on exit**	98.5 (95.1–100)	0.83	−0.003	98.1 (95.1–100)	0.54	−0.008
**Know to take 3 tablets & has tablets on exit or took SP by DOT**	99.0 (95.2–100)	1.0	0.0001	98.6(95.2–100)	0.81	−0.004
**Told when to return for next dose**	14.8 (0–30)	2.53	0.027	15.4 (0–30)	1.87	0.018
**Told to return in 4 to 5 weeks**	79.75 (0–100)	0.58	−0.007	82.7 (0 100)	0.67	−0.006
**Offered an ITN**	42.59 (0–67.86)	10.33	0.164	30.7 (0–67.9)	27.7	0.524

Notes:

CSCom, Community Health Centre; CSRef, Reference Health Centre; N = sample size; DE, Design Effect; ICC, intra-class correlation, SP, Sulphadoxine-pyrimethamine; DOT, Direct Observed Treatment; ITN, Insecticide Treated Nets.

The design effect and intra-cluster variation were generally higher for the CSRef plus CSComs than for the CSComs alone but the pattern was similar across the indicators of intermediate process effectiveness.

## Discussion

This cross sectional health facility survey enabled an in-depth investigation of the ANC experience and interactions with health providers of pregnant women attending ANC at two levels of the health system in Segou District. Using a structured checklist it was possible to quantify these processes and interactions for all women attending ANC on the day of the survey. This quantification of processes plus information collected on characteristics of pregnant women and the health facilities enabled an assessment of a wide range of possible predictors of the effectiveness of these delivery processes.

Delivery of IPTp-SP by DOT was found to be ineffective amongst pregnant women attending ANC for their first or second visit during the current pregnancy, who were of eligible gestation according to national policy. When the requirement of DOT was excluded from the definition of effective delivery of IPTp-SP in a second analysis (a pregnant woman was given the correct number of tablets and had knowledge on how to take them, thus giving the opportunity for her to take an effective dose) the proportion of pregnant women being given a course of IPTp-SP (first or second dose) was higher than for delivery by DOT alone (55.6% and 66.7%) for the CSRef and CSComs respectively, close to the previous Abuja target (60% of 2-dose in 2005) [29], and way below the 100% target for universal coverage [30]. In addition, this was the maximum effectiveness possible as it assumed that 100% of women who had the tablets and the knowledge complied with the instructions given. In practice, this is unlikely to be the case and these are likely to be substantial overestimates. We are not aware, however of any empirical data on the proportion of women who, given tablets of SP for IPTp to take at home, comply with this preventive treatment regimen. Although there is a wealth of data available on the lack of adherence to anti-malarial treatment regimens in the household, as this data is primarily for treatment of symptomatic malaria cases, it is not appropriate to draw parallels here.

After determining that delivery of IPTp-SP was ineffective overall, we were able to identify the specific ineffective processes through an intermediate process analysis. The first of these ineffective processes was that of a pregnant woman being given any SP in ANC consultation. The adjusted predictors of being given SP, with the exception of being second or third trimester, differed between the CSRef and the CSComs. Visit number did not remain predictive of being given SP in the CSRef after adjusting for other potential predictors. The relationship between number of visits and being given SP may have been influenced by attendance of women at more than one health facility. Although we did not assess parity directly we found number of children was not a predictor of being given SP in this setting. Parity has previously been reported as a predictor of receiving IPTp-SP [31], [32]. The finding from other studies which were population based rather than amongst attendees at health facilities, may have been due to lack of adjustment for factors influencing attendance at ANC. More specifically the findings from these studies may have been confounded by earlier attendance at ANC by primigravidae and a greater proportion of primigravidae attending ANC twice.

In the CSRef, having symptoms of malaria, including fever, shivers, or chills reported by the pregnant woman, reduced the odds of being given SP. This may account for visit number not being predictive of being given SP in this health facility. According to the national policy at the time of this study pregnant women with malaria should be treated with quinine, and therefore not being given IPTp-SP was appropriate. Perhaps the most important factor to note here is that as mentioned in the methods, treatment of pregnant women for malaria was not accounted for in the indicator for being given a dose of IPTp-SP used in this study, as we based the analysis on national guidelines which are in-line with the global indicator for assessing coverage with IPTp-SP as measured through the DHS, MIS and other national household surveys. These findings suggest that in Segou District it was important to take pregnant women treated for malaria out of the denominator when assessing effectiveness of delivery of IPTp-SP. According to the structure of the health system in Mali, the main role of the CSRef within the district is to function as a referral centre for patients from the CSComs [33]. It is not surprising therefore that a high proportion of pregnant women attending ANC at the CSRef complained of an illness at the time of their routine ANC attendance (20%). However, the proportion of pregnant women attending ANC with symptoms of malaria in the CSComs was also quite high at approximately 13%. There is a need to assess whether adjustment to the denominator of the delivery and coverage indicator for IPTp-SP is necessary in other settings, and in order to do this the proportion of pregnant women accessing ANC who are treated for malaria should be estimated. The proportion of pregnant women treated for malaria upon attendance at ANC is not currently included in the standard survey tool for the DHS or MIS (http://www.measuredhs.com/What-We-Do/Survey-Types/DHS-Questionnaires.cfm; http://www.measuredhs.com/What-We-Do/Survey-Types/MIS.cfm ) however the proportion of pregnant women who took any anti-malarial drug is often included. More work is needed to understand the relationship between taking anti-malarials for treatment during pregnancy, receiving the required number of doses of IPTp-SP, and how to combine or interpret both indicators.

A further predictor of being given SP in the CSRef after adjusting for other potential predictors was having paid at least CFA 500 whilst in the facility. According to the national policy IPTp-SP should be delivered to eligible pregnant women free of charge [21], however on first visit to ANC there is a fee of CFA 500 for registration. In the CSRef 93% of first visit pregnant women paid CFA500 at registration, 8% of pregnant women on their second visit also paid this fee and 1% of women on their 3^rd^ visit. In addition to registration fees all women attending the CSRef paid CFA 300 for ANC consultation. Both paying CFA 500 in registration and paying for consultation were perfectly predictive of getting SP.

For pregnant women attending the CSComs, educational level together with gestation and visit number was predictive of their being given IPTp-SP. Higher levels of education have been reported as predictive of receiving IPTp-SP [34], [35]; however these studies were population based cross sectional surveys, where this finding may be confounded by increased attendance at ANC amongst those with higher education levels. In such surveys although predictors are adjusted for the influence of other potential predictors these analyses are usually conducted with a denominator of all pregnant or recently pregnant women sampled, rather than amongst women who attended ANC. This access factor may also contribute to parity being reported as a predictor of being given IPTp-SP in population based studies, as mentioned above. It is important in future studies that access to health services and delivery of interventions are disaggregated in predictor analyses.

Unlike the CSRef, paying for registration and paying for consultation were not predictive of getting SP at CSComs; though having spent any money during the visit was a predictor. These findings suggest differences in the way payments are made by women for different procedures and drugs within the CSRef and the CSComs which may in turn relate to the government rather than community funding of the CSRef. However, it is clear that if we are to improve delivery of IPTp-SP in an equitable manner then these cost issues need addressing so that delivery is truly free at all levels of the health system. This predictor offers direct action for a solution to systems ineffectiveness where as other predictors such as palpation do not provide a clear course of action as there are many reasons why this should be predictive of being given IPTp-SP and qualitative data would be useful to provide clarity.

The second ineffective process was giving IPTp-SP by DOT. Delivery of IPTp-SP by DOT was not generally practised in the CSRef. Just one pregnant woman was given a dose of IPTp-SP as directed by the national policy guidelines when attending the CSRef and just one third of those who attended the CSComs were given IPTp-SP by DOT. Amongst pregnant women who were given IPTp-SP in ANC it is not clear why the total amount of money spent in the health facility was a predictor of DOT after adjusting for other potential predictors. This finding suggests that additional approaches are needed to gain a deeper understanding of the processes within health facilities and factors that influence giving treatment by DOT. It has previously been shown that giving IPTp-SP by DOT is influenced by the availability of clean drinking water [36]. Such factors are not easily captured using quantitative approaches and require the addition of qualitative methods to further elucidate and explain quantitative findings. These findings are explored and presented in the companion paper (Webster et al Unpublished).

The delivery of ITNs was ineffective in the CSRef because ITNs were out of stock, and relatively effective in the CSComs. In the CSRef the ineffectiveness was due to a stock-out of ITNs during the period of the survey. Although still below 80% in the CSComs if the 2 CSCom with stock-outs of ITNs during the survey are excluded from the analysis, the proportion of pregnant women who were given an ITN on their first visit to a CSCom was 81.7% and therefore using our definition of 80% of women completing the process, the delivery was effective where ITNs were in-stock. These data suggest that in this setting, if the health facilities have stock of ITNs then, the delivery of them is effective. This assumes that effectiveness of delivery of ITNs in the CSRef would mirror that of the CSComs. This is the first time as far as we are aware that the process effectiveness of direct delivery of ITNs through ANC has been assessed, despite the acknowledged strategic importance of this delivery channel for ITNs [15]. Other studies have evaluated the outcomes at the population level of delivery of ITNs through ANC [37], but have not assessed whether women who attend ANC are offered or are given ITNs.

The estimations of design effect and intra-cluster correlation were important in this study for reasons which can be divided into those relating to internal validity of the study, and those relating to interpretation of the findings for prioritising interventions, and for optimising the design and targeting of these interventions. With this study design and in this setting, clustering by health facility varies greatly by indicator, elevated design effects have been reported in other studies from Benin [38] and Ghana [39]. With a high level of clustering the precision of the estimate for each indicator is reduced as is the power to identify predictors of these indicators. This may have resulted in some predictors being missed in this study. Where the design effect and intra-cluster correlation are high it would not be feasible in terms of the resources required, to achieve the sample sizes needed for high precision on the estimates using this study design. Increasing the number of clusters, that is health facilities, and reducing the number of observations within each facility would be a more operationally feasible approach. However, given the magnitude of the design effect on some of the indicators in this study, the increase in clusters required would also not be achievable.

The calculation of design effects and intra-cluster correlation however, provide some insight into the variability in implementation between health facilities which itself is important and potentially useful information. For example, the two main ineffective processes identified in this study, being given any SP in ANC, and being given SP by DOT are very different in terms of clustering by health facility, with clustering much higher for DOT. This translates to there being a problem in pregnant women being given any SP for some women in many of the health facilities, whilst for being given SP by DOT; the problem is for most women in some health facilities.

The study had several limitations. Non-participant structured observations were the main tool for identifying ineffective processes in this study. The Hawthorne effect is a well recognised limitation of studies where behaviours are observed [40], [41] and it is possible that both the health workers and the pregnant women involved in this study may have changed their behaviours. We assumed that any change in behaviours was towards ‘best behaviour’. The number of observations of some of the health workers involved would have been sufficient for any behaviour change to be likely to revert back to normal during the period of the study [41], but not for the pregnant women. However, given the poor findings on the cumulative and intermediate delivery processes we do not believe that the Hawthorne effect is a substantial worry in interpreting the findings of the study [42]. The method used for enrolling pregnant women was based upon feasibility of implementation of the study and was not technically random. It is possible that the study was biased towards women attending at certain times of day. However, this potential bias did not impact upon the findings as time of attendance was not a significant predictor in univariate analyses of receipt of being given SP or an ITN. It is also possible that health worker behaviour on the busy days chosen for implementation of this study may have differed on less busy days. The study did not include data on the timing since the last dose of IPTp-SP. International and national guidelines state that there should be at least one month between doses of IPTp-SP. In this study therefore we may have over-estimated the health systems effectiveness of delivery of IPTp-SP if women re-attended for a second dose less than one month after receiving the first dose. The inclusion of pregnant women with symptoms of malaria in the denominator may have resulted in an under-estimate of the effectiveness of delivery of IPTp-SP where it is reasonable that these women should be treated for malaria using the national case management guidelines. Assessment of predictors of effectiveness may have been compromised where there were stock-outs of ITNs.

Although we collected information on the health workers involved in the delivery of ANC in the health facilities involved in the study, the individual health workers observed at each stage of ANC were not recorded, only the cadre. This was a limitation to the inclusion of health worker factors in the assessment of predictors of the effectiveness of intermediate processes. However, cadre was not a significant predictor of being given IPTp-SP or an ITN in the univariate analyses.

The strength of this study design is in allowing the in-depth study of health systems delivery processes on the day that a pregnant woman or patient attends the facility. It is not an appropriate study design for providing estimates of the proportion of pregnant women who are given one or more doses of IPTp-SP, during their pregnancy, at the population level. Household surveys are the most appropriate method for this. However, unlike in our health facility study, household surveys rely on self reports from pregnant women. A recent study from Uganda on the validity of pregnant women’s reported behaviour on taking doses of IPTp-SP showed such data to be inaccurate when assessed against plasma levels of sulphadoxine [43].

The fieldworkers in this study were rigorously trained. There are potential limitations to the use of this structured observation method for assessing the effectiveness of delivery of interventions if fieldworkers are not given rigorous training and the tools are not extensively piloted. The two main reasons that the implementation of this method is difficult are firstly that observers need to both look and listen to what is happening in real time. And secondly, unlike the administration of a questionnaire, observers are not able to control the pace of the process if they are unsure of either an action or a verbal interaction between the health provider and the pregnant woman. Rigorous training and extensive piloting are key to achieving quality implementation, together with tailored structuring of the checklists to match the order of processes within the health facilities.

The ultimate use of the findings of this study is to identify reasons for reduced effectiveness in order to improve programme delivery. Quantitative approaches however, are limited in their ability to provide explanations of *why* a process is not working or behaviour is not happening. These quantitative approaches are also limited by their structured nature for example in the CSRef whilst 39 women were observed to have been given tablets of SP during consultation 4 of these women did not have the tablets on exiting the health facility. The reasons for this may be either that the women had taken the tablets after leaving ANC consultation but before exiting the health facility, or that they disposed of the tablets in the health facility. However, as these behaviours were not pre-empted and included in the checklist or exit interview questionnaire, the explanations were not captured. The limited number of predictors of receiving IPTp-SP by DOT in this study is another example where qualitative approaches are needed to understand why this intermediate process was ineffective. For this reason a qualitative study of the reasons for ineffective delivery was explored from the perspectives of health workers involved and the quantitative and qualitative findings used together to identify disorders in implementation and offer practical solutions. These findings are presented in a companion paper (Webster et al Unpublished).

The time-frame and the resources required for such a research study mean that whilst important for identifying implementation problems and for identifying the areas which need further illumination, this kind of approach is not applicable for routine feedback to inform programme implementation and adjustments required to improve its effectiveness. This requires the application of findings from such research studies as this one to adapt routine programmatic monitoring data at the district level for improving the effectiveness of national and sub-national programmes.

In conclusion, in Segou District, the delivery of IPTp-SP was ineffective whilst ITN delivery was ineffective at the district level where ITNs were out of stock and effective in the community level health facilities where ITNs were in stock. The specific intermediate processes which are effective may be identified through quantitative analyses. Regression analyses may be used to successfully identify major predictors of the effectiveness of these processes, but requires additional qualitative analyses to further illuminate factors influencing the delivery processes. Adaptation of this methodology to routine monitoring systems is required to use the opportunity presented by this approach to influence successful uptake of IPT-SP and other interventions at the programmatic level.
